# The sound sensation of a pure tone in cochlear implant recipients with single-sided deafness

**DOI:** 10.1371/journal.pone.0235504

**Published:** 2020-07-13

**Authors:** Jeremy Marozeau, Dan Gnansia, Marine Ardoint, Christine Poncet-Wallet, Diane S. Lazard

**Affiliations:** 1 Hearing Systems Group, Technical University of Denmark, Lyngby, Denmark; 2 Clinical and Scientific Research CI, Oticon® Medical, Vallauris, France; 3 Hôpital Rothschild, Paris, France; 4 Institut Arthur Vernes, ENT Surgery, Paris, France; 5 Institut de l’Audition, Paris, France; University of Melbourne, AUSTRALIA

## Abstract

Ten cochlear implant (CI) users with single-sided deafness were asked to vary the parameters of an acoustic sound played to their contralateral ear to characterize the perception evoked by a pure tone played through the direct audio input of their CI. Two frequencies, centered on an apical and a medial electrode, were tested. In six subjects, the electrode positions were estimated on CT scans. The study was divided in 3 experiments in which the parameters of the acoustic sound varied. The listeners had to vary the frequency of a pure tone (Exp.1), the center frequency and the bandwidth of a filter applied to a harmonic complex sound (Exp.2), and the frequency of the components and the inharmonicity factor of a complex sound (Exp.3). Two testing sessions were performed at 3 and 12 months after activation. The mean results of Exp. 1 showed that the frequency of the matched tone was significantly lower for the apical than for the medial stimulus. In Exp.2, the mean center frequencies of the filters were also significantly lower for the apical than for the medial stimulus. As this parameter modifies the energy ratio between the high and low-frequency components, this result suggests that the medial stimulus was perceived with a brighter timbre than the apical stimulus. In Exp.3, the mean frequencies of the components were not significantly different between the sounds resulting from the stimulation of the two electrodes, but were significantly lower at the12-month session compared to the 3-month visit. These results suggest that a change in place of excitation may be perceived as a change in timbre rather than a change in pitch, and that an effect of adaptation can be observed.

## Introduction

Cochlear implants (CI) restore auditory perception in severely and profoundly deaf patients by bypassing the deficient auditory cells and by electrically stimulating the auditory nerve. Over the years, technological upgrades and new coding strategies have improved speech perception and overall sound quality. Although CIs are widely used and can successfully restore speech perception, it is still unclear how the electric hearing sounds.

Vocoders have been developed to mimic the information delivered to CI users. Simulations with less than eight channels presented to normal-hearing listeners provide speech intelligibility scores in the same range as CI patients [1], [2]. Despite this good result, some researchers argue that the vocoded information does not offer the same sound quality as that of CIs and suggest the existence of perceptual and informational discrepancies between CI stimulation and performance-matched acoustical simulations [3–6]. Thus, a similar level of performance obtained for both real and simulated CI may hide different patterns of errors, limiting the validity of acoustic simulations through vocoders to evaluate new coding strategies. Recently, Dorman *et al*. [6] have demonstrated that CI listeners with single-sided deafness rate vocoded speech presented to their normal ear as very different from the percept of unprocessed speech presented to their CI. Three out of the eight participants evaluated speech processed through a combination of bandpass filters and spectral smearing very similar to the electric percept.

Some studies have tried to match the perception evoked by a single pulse train with an acoustic sound played to the non-implanted ear. Most studies focused on pitch perception using a pure tone as the acoustic reference in CI patients with residual hearing (bimodal rehabilitation) or normal hearing in the non-implanted ear (single-sided deafness) [7], [8], offering a valuable insight on the effect of mismatch between the frequency allocation table of the CI processor and the actual placement of the electrode-array along the cochlea. However, in the late 70s, Eddington *et al*. [9] suggested that the sound sensation of an electric stimulation was rather a complex sound than a pure tone. Recently, this hypothesis was tested in CI users with residual hearing in the non-implanted ear [10]. By modifying the fundamental frequency (F0), bandwidth, center frequency, and the inharmonicity of the acoustic stimulus, it was concluded that the percept given by the stimulation of a single apical electrode did not correspond either to a white noise or to a pure tone, but more to an inharmonic complex signal. However, the “reference” ear being impaired (average hearing thresholds between the listeners: 65 dB at 500 Hz), the matched acoustic sounds may have been distorted. With the emergence of patients implanted with a normal or near to normal ear on the contralateral side, we had the opportunity to reproduce and extend this latter result to a more basal electrode.

Understanding how CI stimulation sounds like is still crucial to improve our understanding of auditory processing differences between CI and acoustic stimulation. Despite many improvements in coding strategies, language comprehension with a CI, especially in noisy environments, remains challenging, as well as gender categorization, emotions perception, and soundscape appreciation. This knowledge will beneficiate to more accurate and realistic CI simulations, helping the design of novel CI processing strategies.

Consequently, our first aim was to find a more precise and realistic acoustic match for the perception of a pure tone processed through a CI in patients with single-sided deafness, i.e. with a normal or near to normal ear used as a reference. Our second aim was to evaluate the change in sound sensation evoked by a shift in frequency of a pure tone processed by the CI. Finally, this study was designed to test the hypothesis that the place of excitation may induce a sensation more related to timbre than pitch [11].

## Methods

In this study, two alternating sounds were presented to the SSD listeners: a reference sound delivered through their CI and a variable sound acoustically delivered to their contralateral ear. They were asked to adjust different parameters of the variable acoustic sound to reproduce the perception of the reference electric sound, and to evaluate their similarity on a visual analog scale.

### Listeners

Ten adults with single-sided deafness participated in this study. They were randomly selected from a sample of twenty-six adults enrolled in a more extensive study about tinnitus treatment based on unilateral cochlear implantation. These 10 subjects were recruited between November 2013 and October 2015. Two centers participated: Rothschild Hospital, Paris, and Ponchaillou Hospital, Rennes. The two projects conformed to The Code of Ethics of the World Medical Association (Declaration of Helsinki) and were approved by the Ethic committee of Comité de Protection des Personnes Ile de France VI (Registration number AFSSAPS: 2012-A014553-40). Each listener enrolled in the present study signed an informed consent form about the main project, and about this supplementary protocol. The experimental design of this study was largely inspired by [10].

**Fig 1:**
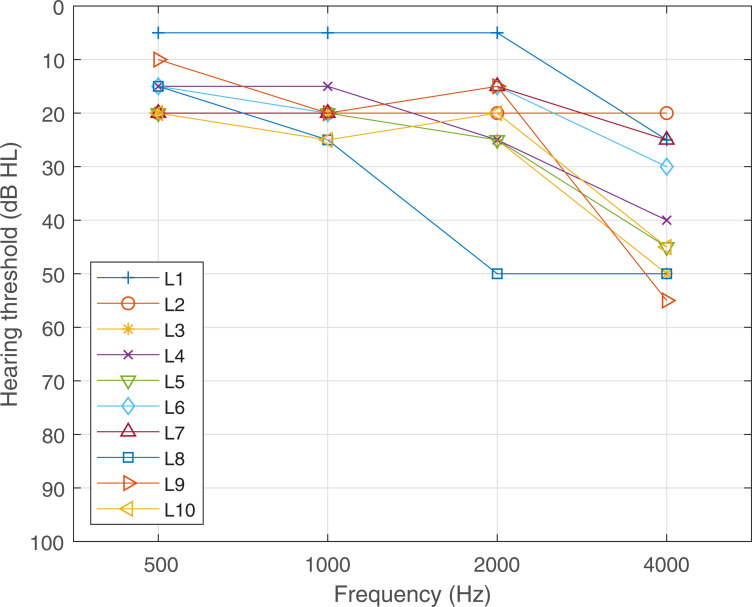
Audiogram of the non-implanted ear of listener 1 to L10.

Subject demographics can be found in Table 1. The average hearing threshold of the non-implanted ear, calculated from the pure tone audiometric thresholds at 500, 1000, 2000, and 4000 Hz, was 24 dB (with a standard deviation of 7 dB; see Fig 1). All the thresholds in the range tested (250–1000 Hz) were lower than 25 dB HL. In the implanted ear, the averaged thresholds were higher than 100 dB HL.

**Table 1:** Listeners’ characteristics and their duration of deafness. Unavailable insertion angle data were estimated by the average of the available data and represented in italic in the table.

Listeners	Gender	Age (y)	Etiology	Estimated duration of deafness (Y)	Insertion angles (^o^)	
					14	20
L1	M	40	Post-middle ear surgery	3.5	329	568
L2	F	54	Sudden deafness–unknown origin	5	286	518
L3	M	70	Sudden deafness–unknown origin	11.8	236	409
L4	M	49	Sudden deafness–unknown origin	10.3	242	424
L5	M	49	Sudden deafness–unknown origin	21	*286*	*490*
L6	M	58	Head trauma–temporal bone injury	7.6	*286*	*490*
L7	M	47	Progressive deafness–unknown origin	1.2	253	432
L8	M	48	Head trauma–temporal bone injury	12.6	*286*	*490*
L9	M	56	Sudden deafness–unknown origin	3.2	*286*	*490*
L10	M	62	Progressive deafness–unknown origin	7	370	592

For six listeners, testing was done after 3 months and 12 months of CI use. One listener performed the three-month session only, and three listeners completed the twelve-month session only. All listeners were users of Oticon® Medical devices (internal part: Digisonic SP EVO, with the Saphyr Neo SP speech processor and Crystalis XDP sound-processing strategy).

### Stimuli

All auditory stimuli were created using the software MAX/MSP (Cycling ‘74®), which also provided the experimental interface and enabled data collection. The CI sound processor was linked to a PC laptop via an auxiliary input direct connection. The electric stimulus was induced by sinusoidal tones. Their frequencies were set to the center of the channel linked to electrode 20 (250 Hz) and electrode 14 (1050 Hz), representing the most apical electrode and a medial electrode of the Oticon® medical device, respectively. These two electric stimuli will be called es20 and es14 from now.

Due to the filter overlap of the sound processor, more than one electrode could have been activated by a single pure tone. Therefore, to test the hypothesis, the input signals were sent to a sound processor, linked to a replica of the electrode array (aka implant-in-a-box, provided by Oticon® Medical) connected to an oscilloscope via a load board. This method showed that only electrode 20 was activated by es20, but that electrode 14 and 13 were activated by es14 (with a ratio of 3 to 2, respectively). It should be noted that this mode of stimulation has the advantage of being safe and reliable for the subjects as no stimulation could exceed the maximum level defined by the patient’s clinical map.

The stimulation rate was the user’s regular rate of 500 pps. Each pulse was composed of an active monophasic and balanced passive discharge using a multi-mode grounding stimulation mode (a combination of 20% monopolar and 80% common ground). The amplitude of the pulse was set to a fixed amplitude (individually set and ranging from 0.74 to 1 mA). The duration of the stimuli varied to adjust the loudness of the stimuli within a range of individualized threshold levels (ranging from 7 to 35 μs) to individualized maximum comfortable levels (ranging from 13 to 56 μs). Acoustic stimuli were presented via an insert earphone (EtymoticH, ER-4P) to the non-implanted ear. The acoustic and electric stimuli shared the same temporal envelope with an overall duration of 900 ms, including a 100-ms ramp up and a 300-ms ramp down in level.

### Procedure

**Fig 2:**
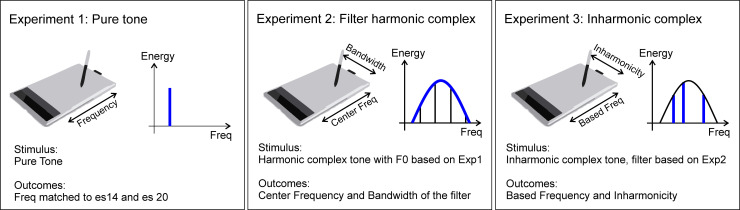
Block diagram of the three experiments. In Exp. 1 (left panel), the participant varied the frequency of a pure tone represented in blue in the Energy/Freq plot. In Exp. 2 (middle panel), the participant varied the center frequency and the bandwidth of a filter (in blue) applied to a harmonic complex sound with a F0 set in Exp. 1, on the two axes of the tablet. In Exp. 3 (right panel), the participant varied the frequency of the components and the inharmonicity factor of a complex sound that was processed through a filter set in the Exp. 2.

The study was divided into three experiments during which different acoustic parameters were varied (see Fig 2). The electric and acoustic stimuli were presented every second. The electric stimulus was fixed, and the acoustic stimulus could be varied by the listeners. Their task was to find the acoustic sound that matched as similarly as possible the perception of the electric stimulus. A graphic tablet (Bamboo Fun pen, Wacom®) was used to adjust the acoustic signal parameters within a two-dimensional space. The position of the pen (on virtual x and y axes) varied the incoming acoustic signal by simultaneously adjusting the values of one to two selected parameters (see below). The parameters selected at the end of one experiment were used to create the stimuli applied to the following experiment, within one trial.

#### Experiment 1: Frequency of a pure tone

The listener was asked to match the frequency of a pure tone (range: 40–2200 Hz) with that of the electric stimulus. The axis (x or y) driving the frequency change varied across trials, the displacement along the other axis did not affect the frequency or any other parameter of the tone.

#### Experiment 2: Harmonic complex tone bandpass filtered

An 11-harmonic complex sound was generated. The phase of each component was randomized. Its fundamental frequency (F0) was the one selected during Exp1 for the corresponding trial. This sound was filtered through a Gaussian bandpass filter symmetrical on the logarithmic scale. One axis controlled the center frequency (CF), ranging logarithmically from F0 to 10 times the F0. The other axis controlled the Q factor of this filter band, ranged logarithmically from 1.4 to 100. The Q factor is defined as the center frequency of the filter divided by its bandwidth (Δf): Q = CF/Δf as well as the slope of the filter with an attenuation of 3 dB for a frequency separation of CF- Δf. Therefore, a high Q value results in a sound with a relatively small number of harmonics, whereas a low Q value results in a more complex sound.

During this experiment, the participants directly manipulated two independent dimensions of timbre. Previous studies [12], [13] have shown that timbre is a multidimensional percept and that each dimension can be correlated with a physical descriptor. One of the first dimension is correlated with the spectral centroid and has been described as brightness. Different descriptors are moderately correlated with the third dimension, but Marozeau *et al*. [13] showed a significant correlation with the spectral spread. In the present experiment, the participants manipulated the latter two dimensions as the CF is directly linked to the spectral centroid and the Q factor to the spectral spread.

#### Experiment 3: Inharmonic complex sound bandpass filtered

An 11-component complex sound with random phases was generated and filtered through the output filter selected at the end of Exp2. One axis controlled the base frequency (Fb) of the sound (range: 40 to 2200 Hz on a logarithmic scale), while the other axis controlled a parameter referred to as inharmonicity, *i*. The composite acoustic signal comprised components with frequencies defined by Fn = Fb*n^*i*^, where Fn was the frequency of the component n (i.e., n was numbered 1–11), and *i* was the inharmonicity exponent, ranging from 0.5 to 2.8 on a linear scale. When *i* = 1 or 2, the sound was harmonic, and Fb was equivalent to a fundamental frequency (F0) that could accurately predict pitch. Values of *i* lower than 1 resulted in a compression of the inter-component frequency spacing whereas higher values resulted in an expansion of the inter-component spacing.

### Protocol

The listeners were all tested in a sound-attenuated booth. First, the presentation level of the electric stimulus was set to be subjectively comfortable by the experimenter. Then the level of the acoustic stimulus was set to a fixed value (in dB SPL) that matched the loudness of the electric stimulus. Before each presentation of an acoustic stimulus, its root-mean-square (rms) value was evaluated and normalized to minimize any potential loudness difference caused by differences in the spectral spread. Furthermore, this level could be modified by the listeners at any moment along with the trials through two physical buttons on the graphic tablet to compensate for any possible loudness difference. Listeners were first familiarized with the interface, and trained during one trial.

Three trials were then recorded, each of them performed in the same order from Exp1 to Exp3, due to their inter-relation (cf above and Fig 2). This sequence avoided any effect of memory for each experiment but could have been sensitive to accumulated errors. However, the frequencies of the tone/components were assessed twice, during Exp1 and Exp3, to minimize this possible effect.

Moreover, to reduce any tendency to return to the same spatial position on the tablet and thereby bias the results, the settings of the tablet were randomly modified by interchanging the axes (x becoming y, and vice versa), and by adding offsets to the origin of the axes (up to 20% shift on each axis) before each trial of each condition. The instructions were to modify the acoustic sound to create a perception similar to that of the perceived electric sensation. There was no time limitation. Subjects had to explore the whole graphic tablet to evaluate the range of acoustic possibilities. When the subjects reached the most satisfying match for one trial, the position of the pen was stored. After each trial of each condition, subjects were asked to rate the similarity between the acoustic sound they had selected and the electric stimulus. Their responses were recorded on a linear scale marked with “completely different” at one end and “exactly the same” at the other. A number between 0 and 10 was assigned to the response, with 10 corresponding to “exactly the same” (visual analog scale, VAS). This procedure was repeated at 3 and 12 months after the first fitting for the two electrodes.

### Determination of electrode positions

Six subjects (L1 to 4, and L7 and 10) had a CT scan after surgery to assess the positioning of the electrode array within the scala tympani (standard procedure of follow up at Rothschild Hospital only, where this subjects were recruited). CT scans were not available for the four other subjects, recruited in the other center. Using these CT images, the estimation of the positions of electrodes 14 and 20 was performed using a method similar to the one described by [14]. First, the center of the round window was manually estimated. The round window position was used to estimate the location of the region of interest (ROI), which is a region of 20x20x20 mm^3^ around the cochlea. A binary image was created by thresholding the image. Voxels with an intensity higher than the top decile among all voxels in the ROI (about 2100 Hounsfield units) were set to the foreground (= 1). A distance map was created from the binary image by computing the Euclidean distance transform (distance between each voxel and the nearest zero voxel). A seed electrode was chosen as the global maximum in the distance map. Then the 19 remaining nodes were determined by growing a graph from the seed electrode. At each iteration, a node was added as the maximal voxel in the distance map that respected the expected distance between electrodes (1.2 mm in the Oticon® medical device). The modiolar axis was estimated using the method introduced by Demarcy *et al*. [15], which models the path of the electrodes with a helical logarithmic spiral. The origin (θ = 0°) was defined by the line joining the modiolar axis and the center of the round window. The angular position of each electrode represented the angle between the origin and the studied electrode. The frequency associated with each electrode rotation angle position was derived from the spiral ganglion frequency map proposed by Stakhovskaya *et al*. [16].

## Results

### Electrode positions

**Fig 3:**
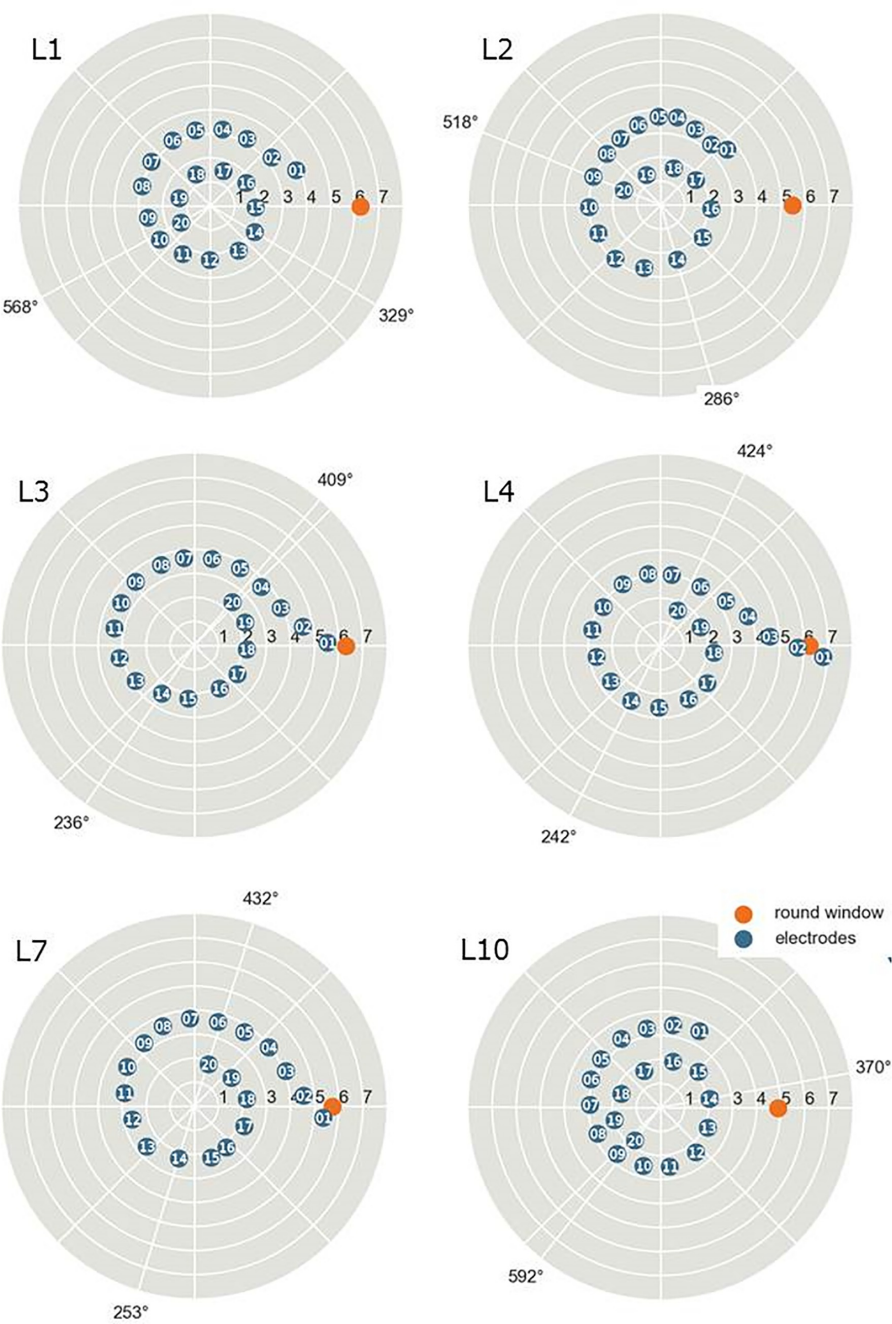
Electrode positions for 6 listeners based on CT scans just after surgery. The two numbers indicate the insertion angle of electrodes 14 and 20.

Fig 3 shows the insertion angle of the 6 listeners with available CT scans. The full insertion depths varied widely among the listeners but always reached at least one full turn. For two listeners, L1 and L10, the most apical electrode reached more than one and a half turns. Electrodes 1 and 2 of L4 were located around the round window and were deactivated by the clinician. The frequency allocation map was only slightly modified in the high frequencies but did not alter the lowest channels (including for the ones associated with electrodes 20, 14 and 13).

### Experiment 1 –Frequency of a pure tone

**Fig 4:**
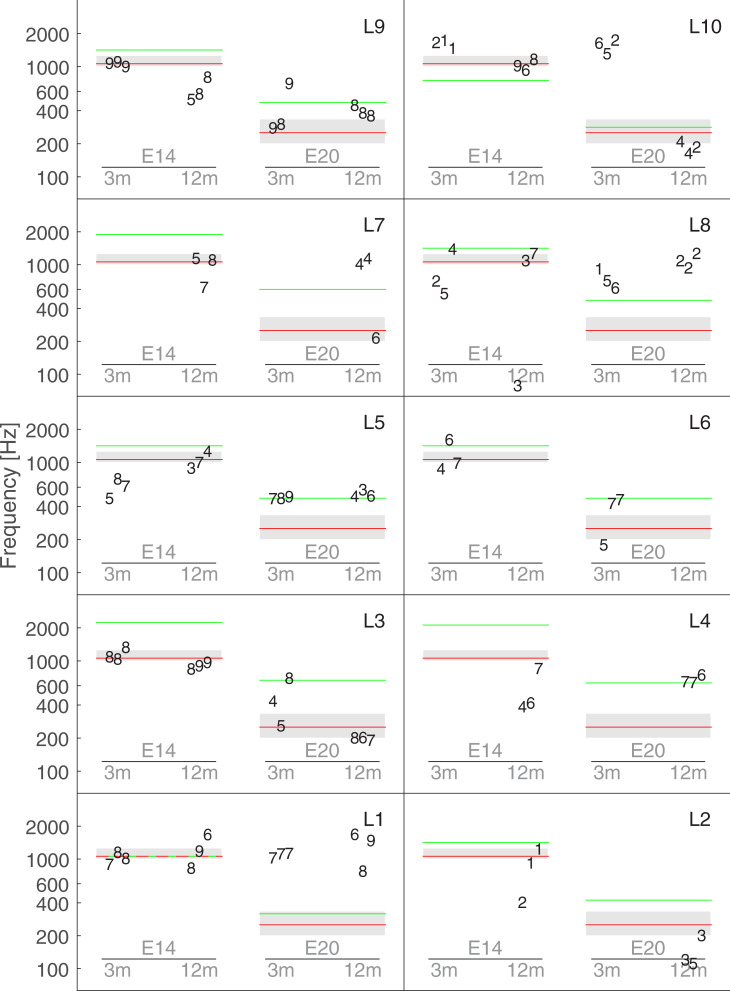
Individual results of Exp1. Each panel represents data from an individual listener. Each data point is represented by the number assigned by the listener on the visual analogue scale (0 corresponding to very different, and 10 to very similar). The red lines indicate the frequency of the reference pure tone processed through the CI (250 Hz for electrode stimulus es20, and 1050 Hz for es14). The spiral ganglion place-frequency prediction is indicated by green lines. The grey boxes outline the acoustic frequency band allocated to each electrode by the manufacturer. This latter was similar for each listener.

Fig 4 shows all the individual matches for the first experiment. Each panel shows the data for one listener. Each data point is represented by the number assigned by the listener on the VAS (0 corresponding to very different, and 10 very similar). For example, L2 was overall dissatisfied with her final matching sound, while L9 thought that the matching sound was very similar to the electric stimulus. Fig 4 shows inter- and intra-subject variability, as well as inter and intra-condition variability. For example, L3 was consistent for the electric stimulus es14 at 3 and 12 months, but showed a less satisfying matching for the electric stimulus es20 at the 3-month session.

The red line indicates the frequency of the pure tone presented to the CI: 250 Hz for es20 and 1050Hz for es14. If a perfect adaptation could occur the frequency of the acoustically matched stimulus would have been similar to the input signal delivered to the sound processor. This correspondence seemed accurate for some subjects such as L1 and L9 for es14 at 3 months, L3 for es14 at both 3 and 12 months and L10 for es14 at 12 months. However, the lack of consistency for the other subjects and conditions makes it difficult to draw a firm conclusion.

By design, each electrode can be activated by a wide range of frequencies. For example, electrode 20 of the sound Digisonic System can be activated for frequencies ranging from 195 to 326 Hz. It has been argued that if adaptation occurs the perception of an electrode array will tend toward the frequency band of its channel (Reiss et al., 2015). In Fig 4, the grey boxes outline the acoustic frequency band allocated to electrode 20 for es20 and to electrodes 14 and 13 for es14. Besides the matched that were centered on the frequency of the input signal (represented by the red line), only a few additional points were located in the frequency band (grey zone), such as L3 for es20 at 12 months. Therefore, it can be concluded that this frequency band is not an accurate predictor of the frequency of the acoustical matches.

Moreover, the frequency range allocated to one electrode is not directly related to its insertion depth. As the cochlea is tonotopically organized, it is possible to estimate the frequency that will acoustically activate the region facing a given electrode. The green lines in Fig 4 indicate the spiral ganglion place frequency based on each listeners’ CT scan analysis and according to a formula derived by Stakhovskaya et al. [16]. Note that for listeners L5, L6, L8, and L9 for whom CT scans were not available, the average insertions derived from the other 6 listeners, i.e., 289^o^ for electrode 14 and 493.5^o^ for electrode 20 were used. For L1, the spiral ganglion place frequency for es14 was very close to 1050 Hz so the green line is hidden by the red one. Only two subjects, L5 and L4, seem to base their matched for es20 to this frequency.

Overall, neither the frequency of the input sound (red lines), the frequency allocation band (grey rectangle) nor the spiral ganglion place frequency prediction (green lines) reliably predicted the collected data.

**Fig 5:**
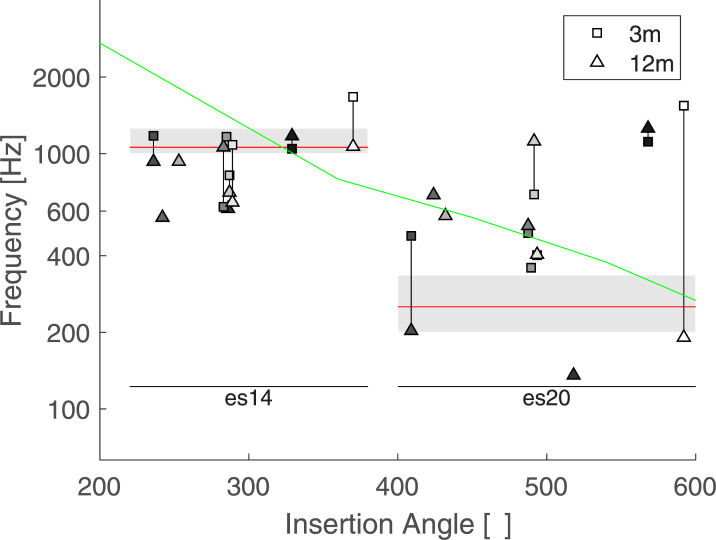
Average results of Exp 1. Each symbol represents the weighted average of one condition for each listener as a function of the insertion angles. Squares represent data collected at the 3-month session and triangle at the 12-month session. Listeners are coded with different shadings. Two data points collected by the same listener are linked by a vertical line. The spiral ganglion place-frequency prediction is indicated by a green line and the acoustic frequency bands of electrodes 14 and 20 are indicated by grey boxes.

Fig 5 shows the averaged results for each listener as a function of insertion angles of electrode 14 and 20, at 3 and 12 months. Each individual measures were weighed by the corresponding VAS value. The data collected at the 3-month visit are represented by a square and the data collected at the 12-month visit by a triangle. A vertical line links two data points collected for the same listener and therefore indicates potential changes with time. The green line indicate the relationship between a given insertion angle and the spiral ganglion place frequency [16]. A mixed linear model was performed including the individual matches as independent variables, the electrodes, sessions, and their interactions as fixed effects and the listeners as random effects. As the insertion angle was missing for 4 listeners, the electrode number was preferred as a fixed factor in the statistical model. Only the main effect of electrode was found significant (F(1,7.369) = 8.5391, p = 0.021). On average, the sensation induced by es20 matched a tone with a frequency of 506 Hz and that of es14 matched a tone with a frequency of 901 Hz. As expected by the tonotopic organization of the cochlea, es20 –the most apical stimulus—matched a tone with a lower frequency than es14. However, the matched frequency was about twice the value of the input signal for es20 (i.e. a 250 Hz pure tone processed though the CI was perceived on average as a 506 Hz tone presented to the normal ear), and about 10% lower for es14 (i.e. a 1050 Hz pure tone processed though the CI was perceived on average as a 901 Hz tone presented to the normal ear). No significant effect was observed for the main factor session (F(1,6.164) = 1.8367, p = 0.2229), nor its interaction with the electrode (F(1,4.951) = 0.2509, p = 0.6379).

### Experiment 2 –Harmonic complex sound bandpass filtered

During this experiment, the listeners were asked to adjust two parameters of a band-pass filter: the center frequency and the bandwidth.

**Fig 6:**
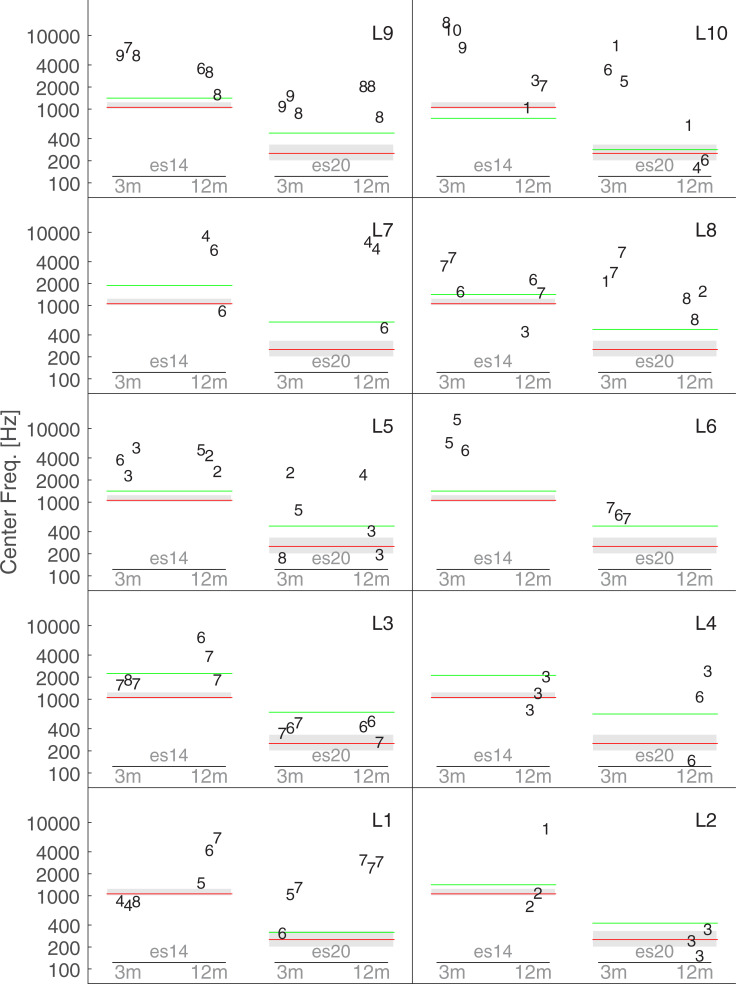
Individual results of Exp2-Center frequency. Individual results for the center frequency of the applied filter. See caption of Fig 4 for more information.

**Fig 7:**
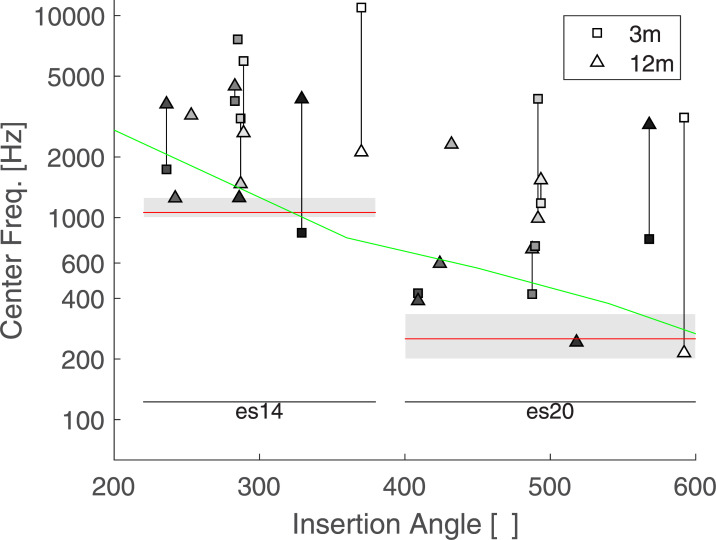
Average results of Exp2-Center frequency. Average results for the center frequency of the applied filter. See caption of Fig 5 for more information.

The individual and averaged results of the center frequency of the filter applied are shown in Figs 6 and 7 respectively. The same representation as in Figs 4 and 5 with an extended y-axis was used. The filter’s average center frequency was 2850 Hz for es14 and 864 Hz for es20. Overall the center frequency of the filter matched was higher by 70 and 170% than the frequency of the pure tone in the first experiment for es20 and es14 respectively. Note that by design, the center frequency could not be set at a level below the value set in the first experiment. A similar statistical analysis as for Exp 1 was run. Likewise Exp1, the effect of electrode was significant (F(1,8.542) = 18.5543, p = 0.002), with no effect of session (F(1,8.694) = 0.92, p = 0.36), or electrode interaction (F(1,7.253) = 0.001, p = 0.97).

**Fig 8:**
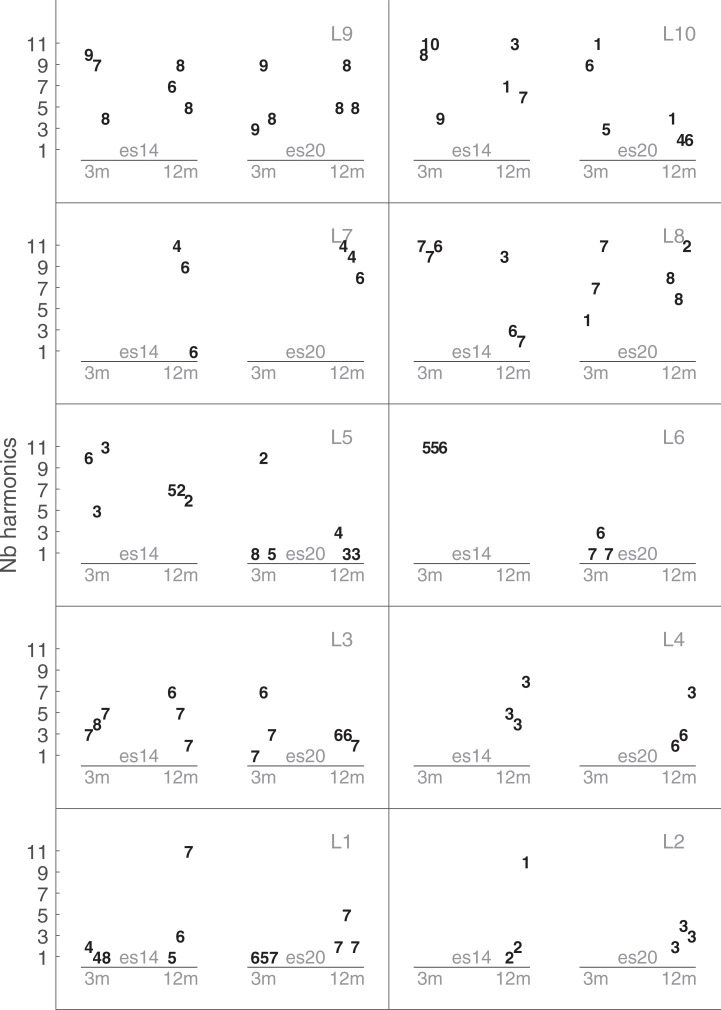
Results of Exp2-Band width. Individual results for the width of the filter defined by the number of perceived harmonics. See caption of Fig 4 for more information.

By adjusting the bandwidth of the filter (Q-Factor), this experiment allowed us to assess whether a pure tone processed through a CI was perceived as a pure tone or as a complex signal. Therefore, it is more relevant to study the effect of the bandwidth by deriving the number of perceived harmonics. This number depends on the Q-Factor, but also on F0 and the center frequency to a lesser extent. The number of harmonics perceived was estimated as the number of components presented in the signal with an attenuation of less than 40 dB compared to the level of the strongest harmonic after the filter. Fig 8 shows these results for each listener. The number of perceived harmonics ranged from 1, a pure tone, to 11, the maximum complexity, and averaged to 5.53. Most of the listeners (L1, L2, L3, L5, L6 and L7) perceived a pure tone during one trial at least. It should be noted that those data might have been biased by a border effect that could have lowered the chance to observe trials with single pure tones (the lowest possible number of harmonics).

The statistical analysis was run with the number of perceived harmonics as the dependent variable. No significant effect of electrodes (F(1,3.891) = 5.8013, p = 0.076), session (F(1,7,545) = 0.4487, p = 0.52), or their interactions (F(1,3.764) = 1,2415, p = 0.3312) was found.

### Experiment 3 –Inharmonic complex sound bandpass filtered

**Fig 9:**
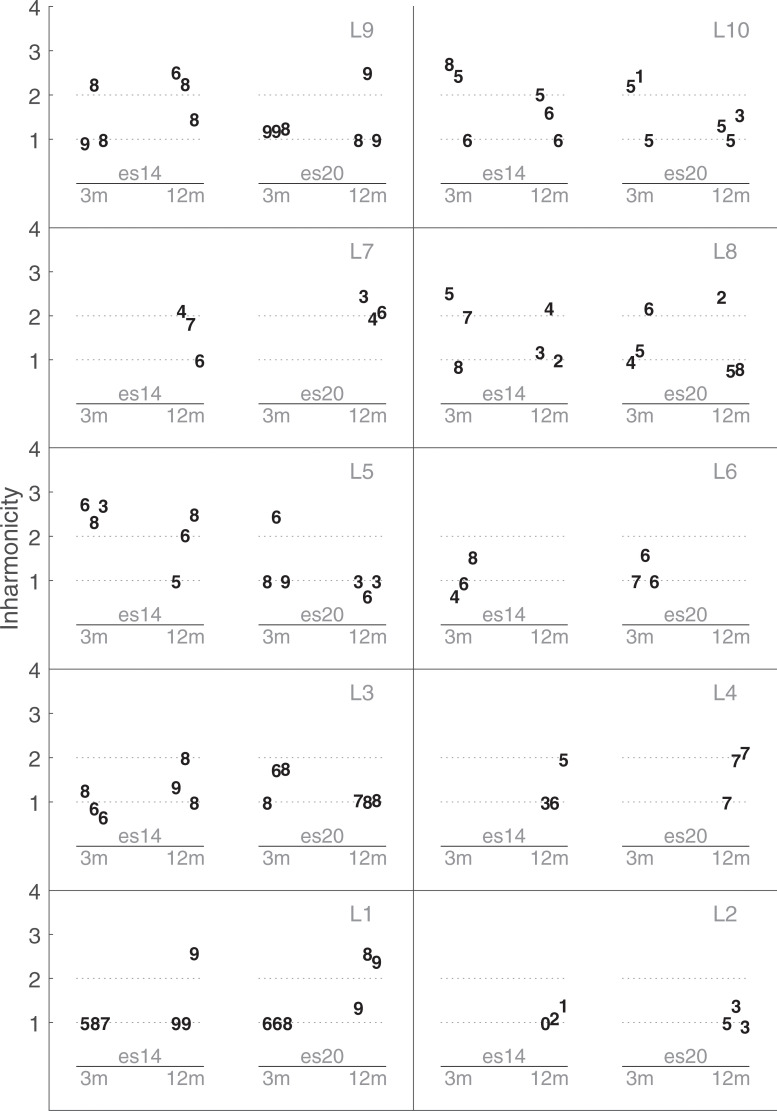
Results of Exp3-Inharmonicity. Dashed lines indicate values for harmonic sounds (1 or 2). See caption of Fig 4 for more information.

Fig 9 shows the individual results of the inharmonicity parameter. An inharmonicity number of 1 or 2 produces a harmonic sound, a value lower than 1 produces an inharmonic sound with compressed harmonics, and a value larger than 1 produces an inharmonic sound with expended harmonics. Fig 9 shows a large inter-listener variability, but on average the inharmonicity number was larger than 1. No significant effect of electrodes (F(1, 7.818) = 0.0143, p = 0.91), sessions (F(1, 8.991) = 0.3768, p = 0.55), or their interactions (F(1, 8.13) = 1.19, p = 0.31) was found.

**Fig 10:**
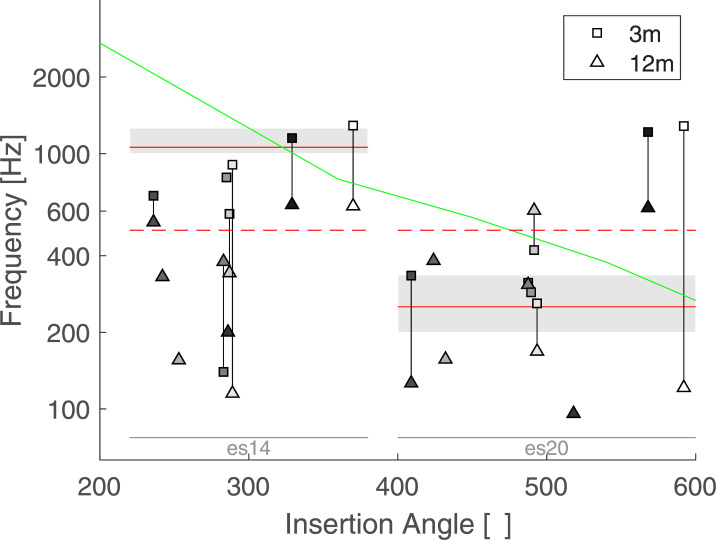
Results of Exp3-Based frequency. Average results for the based frequency of the applied filter. The dashed line indicates the clinical pulse rate of 500 Hz. See caption of Fig 5 for more information.

Fig 10 shows the averaged results for the based frequency parameter. As the matched sounds were mostly inharmonic, one should be careful not to interpret this factor as a predictor of pitch. Overall the based frequency (Fb) was lower than the tone frequency found in Exp 1, and the center frequency found in Exp 2 (Fb = 433 Hz and 307 Hz for es14 and es20, respectively). The averaged values for es14 were mostly lower than the spiral ganglion place-frequency prediction and the frequency allocation band, but were slightly lower than 500 Hz. Unlike the frequency in Exp 1 and the center frequency in Exp 2, Fb was not significantly affected by an electrode effect (F(1, 8.207) = 3.4994, p = 0.0974). However, an effect of session was observed, with a lower Fb for both electrodes between 3 and 12 months of CI use (F(1, 7.042) = 6.7421, p = 0.035), with a Fb of 552 Hz at 3m and 264 Hz at 12 months. No significant interaction was found (F(1, 8.572) = 0.0335, p = 0.8589).

VAS similarity ratings were analyzed using a mixed linear model, with listeners as a random factor, and electrodes, experiments and time-points as fixed factors. No significant effect was found.

## Discussion

Despite the increasing number of CI recipients, the perception of the electric sensation is still unclear, and an acoustic definition is lacking. Our sample of subjects with an implanted ear and near to normal contralateral ear offered a unique opportunity to compare electric and acoustic stimulation within the same subject. We proposed in the present study to characterize the perception of the electric sound in more parameters than just pitch, on an apical and a medial electrode.

### Pitch matching

During the first experiment (Exp 1), the listeners were asked to adjust the frequency of a pure tone presented at 250 and 1050 Hz to the CI. The 250 Hz pure tone (es20) activated the most apical electrode only (electrode 20), while the 1050 Hz pure tone (es14) activated electrode 14 and 13. Such tasks have been performed in previous studies [7], [8], [17–22]. However, in these studies, the pulse rate was set to a level either below 40 or above 700 pps in order to minimize the temporal pitch cues. In the current experiment, we intentionally decided to keep the usual listener’s clinical rate of 500 pps, as the overall goal of the study was to characterize the “realistic” sound quality of a pure tone, and not to study place pitch *per se*. This difference in parameters makes the comparison difficult with the previous studies. Indeed, as normal-hearing listeners perceive a complex signal with the different dimensions of chroma, tone height (or brightness) and pitch strength, one could argue that CI users might perceive the electric stimuli as a complex pitch composed of more than one dimension [23]. The results of Exp 2 and Exp 3 support this hypothesis. In these latter experiments, the listeners adjusted the center frequency of the filter on average to a much higher value than the based frequency of a complex tone. It may be assumed that these two parameters are related to two different perceptual dimensions of pitch (or timbre) (as suggested by Tong *et al*. [24]). Under this assumption, varying the rate of a stimulus while keeping the same electrode position should result in the perception of an acoustic stimulus with the same CF (Exp 2), but with a different Fb (Exp 3).This hypothesis needs to be tested.

### Brightness and tonality

In Exp 2, the average center frequencies of the filters matching the CI users’ perception of es20 and es14 were 864 Hz and 2850 Hz, respectively. This parameter was designed to assess the relationship between the low and high frequencies of the spectrum. A low center frequency produces a sound with mostly low frequency components possibly perceived as “dull.” Conversely, a high level of this value may be perceived as “bright.” In fact, the center frequency of a symmetrical spectrum can be considered a physical descriptor of the perceptual dimension of brightness [12]. Therefore, the result of Exp 2 suggests that a pulse train delivered to electrodes 14 and 13 was perceived with a brighter timbre than the same pulse train delivered to electrode 20. In Exp.3, the frequency of each component was set by the based frequency and the inharmonicity factor. As the resulting sound was inharmonic, the based frequency could not accurately predict pitch. Taken together however, based frequency and inharmonicity influenced the tonality of the sound, which may be related to chroma and pitch strength rather than to timbre. Finally, while no significant effect of electrode position was found, a significant effect existed for the center frequency in Exp 2. This result supports the hypothesis that electrode position along the cochlea induces a perceptual change that is more similar to a change in timbre (brightness) than to a change in pitch, as experienced by normal-hearing listeners [25].

### Effect of adaptation

The experiments were performed at 3 and 12 months after the first fitting to monitor any effect of adaptation. It has been previously argued that the sound sensation induced by the electric stimulation is influenced by the anatomical location of the electrodes along the cochlea just after implantation and then adapts to the frequency band allocation with time [8, 26]. However, these studies showed very idiosyncratic results: some listeners adapted easily, while some did not at all [20]. No indicator predicting the level of adaptation for one individual has been found so far. In the present study, an effect of time was found only for the based frequency in Exp3 and at the group level. On average, the based frequency decreased between the two testing sessions. Neither the frequency allocation, the spiral ganglion frequency map, nor the pulse rate (500 pps) reliably predicted the based frequency, possibly because these factors do not account for the residual neural population nor for the distance between the electrode and the modiolus. The lack of effect of time observed in Exp 1 and Exp 2 may be explained by a possible rapid adaptation of SSD listeners within the first 3 months, or that no adaptation occurs.

### Effect of spread of activation

The electric stimuli were generated by a tone with a specific frequency sent through the auxiliary input. As the frequency band analysis of the sound processor allows for some overlap, a simple pure tone can potentially activate more than one electrode. Given the frequencies selected in this study, we verified that the 250 Hz pure tone activated electrode 20 only. However, the 1050 Hz pure tone activated electrode 14 primarily, but electrode 13 also to some extent. Therefore, this spread of activation may have influenced the final match of the three experiments. In Exp 1, it may have induced a higher pitch or a sensation averaging electrode 14 and 13 [27]. In Exp. 2, it may have induced a larger number of perceived harmonics for es14 compared to es20, and in Exp. 3, a larger inharmonicity factor. However, in the latter two cases, no significant effect of electrode was found.

### Pitfalls and possible improvements

This study aimed to create an acoustic equivalent of the sensation of a pure tone perceived through a cochlear implant. As the possible variation of an acoustic sound is almost infinite and considering that the listeners were not accustomed to creating sounds, a trade-off had to be made between the degree of freedom that the listener had and the acoustic parameters. As those choices may have affected the final match, it is essential to consider the results within this framework.

#### Loudness

At the beginning of each trial (three in total), the listener had first to adjust the level of both stimuli to an equivalent comfortable loudness. However, as the listener modified the spectral property of the sound, the loudness may have been affected, through a difference in frequency or spectral spread. To minimize this effect of loudness, the root mean square value of the acoustic stimulus was set to a value that the listener was encouraged to adjust through two side buttons at any moment during the trial. However, this methodology could not have prevented some small loudness variations of the acoustics stimulus not corrected by the participant. Therefore, the participant may have been biased toward a sound that had a loudness more similar to the implant sound, instead of focusing purely on sound quality. This point may be improved in the future by using a loudness model keeping the loudness constant.

#### Order of the three steps

This study aimed to partially replicate the data from Lazard et al. [10], and use therefore a similar methodology. In this approach, the listener needed first to adjust a pure tone to match the percept of a possible complex sound induced by the CI. In the second stage (Exp 2), the listener adjusted the center of a filter that could only be above the frequency set during the first stage (Exp 1). Therefore, it may be possible that this procedure had induced an overestimation of the center frequency. To improve this possible bias, the order of the experiments could be reversed in a future study: first, the participant would adjust the center frequency of a low-F0 harmonic complex tone, then she/he would modify the F0 of a filtered complex sound.

#### Feasibility and rating of the match

Unlike the study of Lazard et al. [10], which found a clear improvement of the similarity rating with the complexity of the stimuli, the rating did significantly differ between the experiments in the present study. Furthermore, as the ratings rarely reached the maximum value (“exactly the same” on the visual analogue scale), one can say that the task was difficult. Thus, without managing to provide a perfect definition, the results of this study adds to the literature about what a sine input sounds like for a CI listeners.

## Conclusions

These three experiments were designed to find an acoustic match for 250 and 1050 Hz pure tones processed through a cochlear implant. The results indicate that the best match was obtained for a complex sound with a bright timbre for both stimuli. Exp 1 indicates that the frequency of the matched pure tone was lower for the apical electrode than for the medial electrode. The matched frequency was stable between 3 and 12 months. Neither the spiral ganglion frequency map nor the frequency allocation band correctly predicted what listeners described. The results of Exp 2 show that the 1050 Hz pure tone (centered on electrodes 14 and 13) was perceived with a brighter timbre than the 250 Hz pure tone (centered on electrode 20). As no effect of electrode position was found in Exp 3 on the frequency of the component or on the inharmonicity factor, it can be concluded that electrode position influences the perception of timbre rather than the perception of tonality of a pulse train.
